# Evolution of *mal *ABC transporter operons in the *Thermococcales *and *Thermotogales*

**DOI:** 10.1186/1471-2148-8-7

**Published:** 2008-01-15

**Authors:** Kenneth M Noll, Pascal Lapierre, J Peter Gogarten, Dhaval M Nanavati

**Affiliations:** 1 Department of Molecular and Cell Biology, University of Connecticut, Storrs, CT 06269-3125, USA; 2 Biotechnology Bioservices Center, University of Connecticut, Storrs, CT 06269-3125, USA; 3 Analytical Biochemistry Section, Laboratory of Neurotoxicology, NIMH, Bethesda, MD 20892-1262, USA

## Abstract

**Background:**

The *mal *genes that encode maltose transporters have undergone extensive lateral transfer among ancestors of the archaea *Thermococcus litoralis *and *Pyrococcus furiosus*. Bacterial hyperthermophiles of the order *Thermotogales *live among these archaea and so may have shared in these transfers. The genome sequence of *Thermotoga maritima *bears evidence of extensive acquisition of archaeal genes, so its ancestors clearly had the capacity to do so. We examined deep phylogenetic relationships among the *mal *genes of these hyperthermophiles and their close relatives to look for evidence of shared ancestry.

**Results:**

We demonstrate that the two maltose ATP binding cassette (ABC) transporter operons now found in *Tc. litoralis *and *P. furiosus *(termed *mal *and *mdx *genes, respectively) are not closely related to one another. The *Tc. litoralis *and *P. furiosus mal *genes are most closely related to bacterial *mal *genes while their respective *mdx *genes are archaeal. The genes of the two *mal *operons in *Tt. maritima *are not related to genes in either of these archaeal operons. They are highly similar to one another and belong to a phylogenetic lineage that includes *mal *genes from the enteric bacteria. A unique domain of the enteric MalF membrane spanning proteins found also in these *Thermotogales *MalF homologs supports their relatively close relationship with these enteric proteins. Analyses of genome sequence data from other *Thermotogales *species, *Fervidobacterium nodosum*, *Thermosipho melanesiensis*, *Thermotoga petrophila*, *Thermotoga lettingae*, and *Thermotoga neapolitana*, revealed a third apparent *mal *operon, absent from the published genome sequence of *Tt. maritima *strain MSB8. This third operon, *mal3*, is more closely related to the *Thermococcales*' bacteria-derived *mal *genes than are *mal1 *and *mal2*. *F. nodosum*, *Ts. melanesiensis*, and *Tt. lettingae *have only one of the *mal1-mal2 *paralogs. The *mal2 *operon from an unknown species of *Thermotoga *appears to have been horizontally acquired by a *Thermotoga *species that had only *mal1*.

**Conclusion:**

These data demonstrate that the *Tc. litoralis *and *P. furiosus mdx *maltodextrin transporter operons arose in the *Archaea *while their *mal *maltose transporter operons arose in a bacterial lineage, but not the same lineage as the two maltose transporter operons found in the published *Tt. maritima *genome sequence. These *Tt. maritima *maltose transporters are phylogenetically and structurally similar to those found in enteric bacteria and the *mal2 *operon was horizontally transferred within the *Thermotoga *lineage. Other *Thermotogales *species have a third *mal *operon that is more closely related to the bacterial *Thermococcales mal *operons, but the data do not support a recent horizontal sharing of that operon between these groups.

## Background

The genome sequence of the bacterial hyperthermophile *Thermotoga maritima *revealed evidence of extensive horizontal gene transfer (HGT) with archaea [1]. Subsequent analyses of its genome sequence along with analyses of large tracts of sequences from other members of the *Thermotogales *have supported and extended this observation [2,3]. Many of the genes that have been shared with archaea encode ATP binding cassette (ABC) transporters. Although originally characterized as oligopeptide transporter genes, analysis of the substrate binding proteins encoded by these operons showed that oligosaccharides are their likely substrates [4]. The *Tt. maritima *genome encodes many other ABC transporters. Indeed, its genome encodes the second highest proportion of ATP-dependent transporter genes (including ABC transporters) among currently sequenced bacterial and archaeal genomes [5]. Some of the *Tt. maritima *transporters have been experimentally shown to encode sugar binding proteins [4] including two proteins that bind maltose [6].

**Figure 1:**
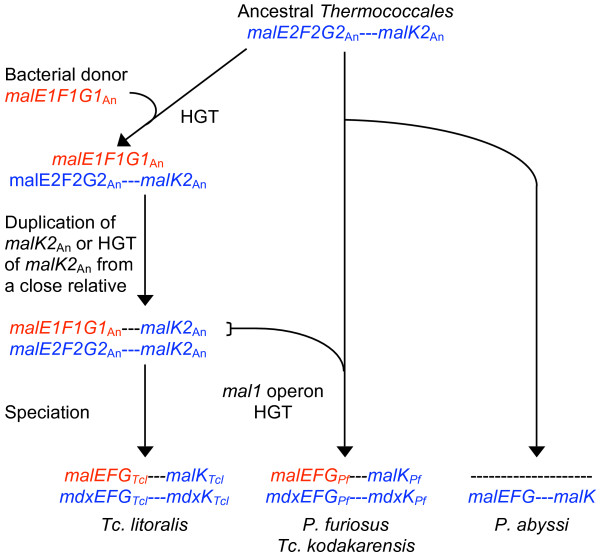
**The evolutionary history of the *mal *genes in the *Thermococcales***. *mal*E/*mdx*E encode the substrate binding proteins (SBPs), *mal*F/*mdx*F and *mal*G/*mdx*G encode membrane spanning proteins (MSPs) also known as permease subunits, and *mal*K/*mdx*K encode ATP binding proteins (ABP) containing an ATP binding cassette (ABC). Subscript An denotes ancestral genes.

Horizontal acquisition of transporter genes also occurred among the hyperthermophilic archaea. A striking example of this is the complex evolutionary history of the *mal *operons in the *Thermococcales *as depicted in Figure 1. While the genomes of *Pyrococcus woesei*, *P. abyssi*, *P. horikoshii*, and *Thermococcus kodakarensis *all encode one *mal *operon, the genomes of *P. furiosus *and *Tc. litoralis *each encode two [7,8]. The second *mal *genes found in each of the latter two organisms (previously designated as the *mal2 *genes, but called the *mdx *genes here, as in [9]) are orthologous to the genes in the single operons of the former organisms. The *mdx *operon in *P. furiosus *(PF1938-1936 and PF1933) encodes a maltodextrin binding protein (MdxE_*Pf*_, PF1938) and is upregulated in response to growth on maltose and starch [10,11]. In *P. furiosus*, the *mal *operon (PF1739-1741 and PF1744) encodes MalE_*Pf*_, a protein that binds maltose and trehalose, but it does not appear to function as its major maltose transporter [10-13]. The orthologous *mal *products in *Tc. litoralis *have been extensively studied [13-17]. The region encoding the *mal *genes are flanked by insertion sequences in both *P. furiosus *and *Tc. litoralis *suggesting these genes were shared between these organisms by HGT [8]. The similarity of the two MalK homologs (the ATP-binding proteins) in *Tc. litoralis *(MalK_*Tcl *_and MdxK_*Tcl*_) suggests that one of them arose by a duplication of the other [18]. Subsequently, the *mal*-linked *malK *transferred to the ancestor of *P. furiosus *along with the entire *mal *operon [18] (Fig. 1).

This history of horizontal transfer of archaeal *mal *genes raises the possibility that bacterial thermophiles that live among these archaea may also have shared these maltose operons. *Tt. maritima *was originally isolated from sediments on Vulcano Island, Italy, the same area from which *P. furiosus *and *Tc. litoralis *were isolated [19-21]. Hamilton-Brehm, *et al*. speculated that because these two archaea lived together, they were more likely to exchange *mal *genes [7], so it is conceivable that bacteria living among them, like the ancestor of *Tt. maritima*, might have acquired those genes, too. There is evidence of transfer of whole operons to the *Thermotoga *lineage from the *Thermococcales*. Most striking, perhaps, is the horizontal transfer of the twelve-gene *mbx *operon encoding an NADH:ubiquinone oxidoreductase complex that is now found adjacent to the *mal1 *operon in *Tt. maritima *[22].

A simple BLAST analysis of the *Tt. maritima *Mal amino acid sequences suggested they are of bacterial origin [1]. The two sets of *Tt. maritima mal *genes are their closest homologs indicating a duplication event or HGT within the *Thermotogales *lineage gave rise to one of them. Other BLAST hits are mainly to bacterial sequences, though some archaeal sequences are also retrieved. Although these data suggest the *Tt. maritima mal *genes are of bacterial origin, no rigorous phylogenetic analysis has been published that rules out the possibility that these apparently bacterial genes originated from archaeal genes and perhaps were a part of the archaeal HGT events described above. This report provides a detailed examination of the evolutionary history of these *mal *genes.

The *Tt. maritima mal *genes are in two operons each containing three, collinearly transcribed genes encoding a substrate binding protein (SBP, MalE) and two membrane-spanning proteins (MSPs, MalF and MalG). However, the *malF2*_*Ttm *_ORF is truncated and contains an authentic frameshift mutation. A short ORF (TM1838) precedes it. Neither operon contains a gene encoding the necessary ATP-binding protein (ABP) that provides the energy for substrate transport. The *mal1*_*Ttm *_operon (TM1204-2) encodes an apparent mannooligosaccharide transporter that also can bind maltose and maltotriose [6]. The *mal2*_*Ttm *_operon (TM1839-36) encodes a maltose and trehalose transporter [6].

Recently, genome sequence data of several other members of the *Thermotogales *have become available. Sequence data from the genomes of *Fervidobacterium nodosum*, *Thermosipho melanesiensis*, *Thermotoga petrophila*, *Thermotoga lettingae *and others are publicly available and a partial genome sequence of *Thermotoga neapolitana *is available from The Institute for Genomic Research. This information from other *Thermotogales *species provides data to examine how the *mal *operons have evolved within this lineage and perhaps can provide evidence about the duplication event that gave rise to the two *mal *operons now found in *Tt. maritima*. In this examination of the deep phylogenetic relationships among the hyperthermophiles' *mal *genes, we also uncovered unreported features of the *Thermotogales *Mal protein sequences that not only enlighten our understanding of their evolutionary histories, but also suggest novel structural or functional features of the transporter proteins.

## Results

### Evolution of the *mal *and *mdx *genes in the *Thermococcales*

Previous sequence comparisons using BLAST analyses indicated that one *mal *operon (the ancestral *mal1 *operon, *mal1*_An_, along with the ancestral *malK2*_An _gene in Figure 1) transferred from an ancestor of *Tc. litoralis *to an ancestor of *P. furiosus*, likely involving a transposition mediated by the insertion sequences now found in *P. furiosus *[8]. Based on a simple comparison of the genes encoding ABPs in the *mal *and *mdx *operons in *Tc. litoralis *with their homologs in *P. furiosus*, a copy of an ancestral *mdxK *gene (*malK2*_An_) was postulated to have recombined downstream of the *mal1*_An _operon in an ancestor of *Tc. litoralis *(Fig. 1), where it now encodes the ABP for the *mal*-encoded transporter [18]. This ancestral *mdxK *could have originated from gene duplication or horizontal acquisition. We performed phylogenetic analysis of all the proteins encoded by the *mal *and *mdx *operons to test these hypotheses and to determine the origins of these evolutionarily mobile genes.

**Figure 2:**
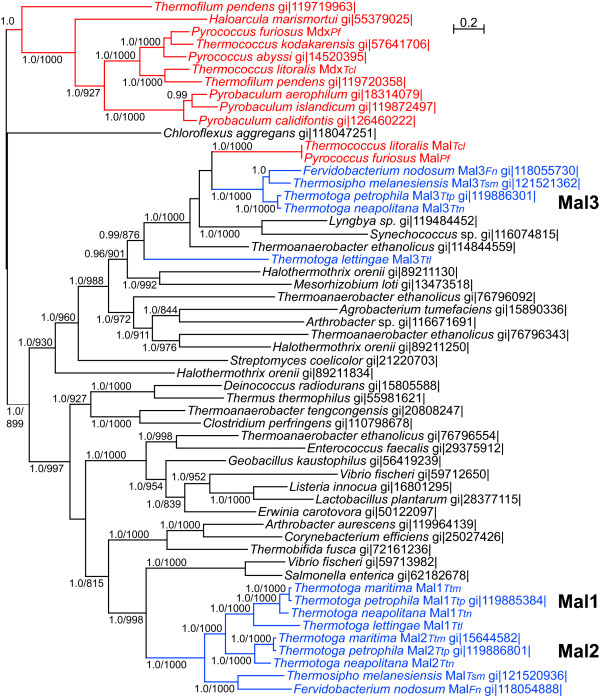
**Relationships among families of apparent maltose ABC transporter operons in archaea and bacteria**. Homologs of the *P. furiosus *MdxE (PF1938) were gathered using that sequence as query in BLASTP. Concatenated sequences of these SBP homologs and their cognate MSP and ABP from each operon were prepared, aligned and analyzed. A ML tree generated in PHYML is shown. Values shown are Bayesian posterior probabilities followed by bootstrap support values calculated with PHYML. Bootstrap support values below 800 and posterior probabilities below 0.8 are not shown. For reference, the gi numbers of the MalE (SBP) sequence for each operon are provided. Archaeal sequences are shown in red, those from *Thermotogales *in blue, and from other bacteria in black. The tree depicted here should be considered unrooted.

The MdxE_*Pf *_(PF1938) protein sequence was used as a query for a BLAST search of the non-redundant protein database at NCBI. The top 250 hits were aligned using ClustalX and then used to construct neighbor joining trees. Using these preliminary relationships as a guide, sequences were chosen from archaea, *Thermotogales *species, and bacteria closely related to the archaeal sequences to represent a broad spectrum of taxa. These selected sequences were then placed in a new database (approximately 60 sequences for each gene). Associated MalF and MalG protein sequences were concatenated with each MalE-like sequence only if these three ORFs were the only ORFs in an apparent ABC transporter operon. These concatenated sequences were aligned and their phylogenetic relationships were examined by maximum likelihood (ML) and Bayesian analyses. The tree constructed from this ML analysis is shown in Figure 2 with ML bootstrap values and Bayesian posterior probabilities for each analysis placed thereon.

The most obvious feature of this tree in regard to the *Thermococcales *sequences is the independent evolution of the archaeal *mal *and *mdx *operons. The distant evolutionary relationship of these two operons is well supported. The Mdx sequences cluster with strong support with those from other archaea while the Mal sequences cluster separate from other archaeal sequences. The latter cluster with sequences from a variety of bacteria including cyanobacteria and members of the *Thermotogales*. These data support the independent evolution of these two sets of genes and suggest that the *mal *genes found in *Tc. litoralis *and *P. furiosus *were acquired from a bacterium by an ancestral member of the *Thermococcales *(Fig. 1). We shall discuss the relationship of these *Thermotogales *operons to the *Thermococcales mal *operons after considering the history of the *Thermococcales malK *genes.

### Evolution of the *malK*/*mdxK *genes and their *Thermotogales *homologs

**Figure 3:**
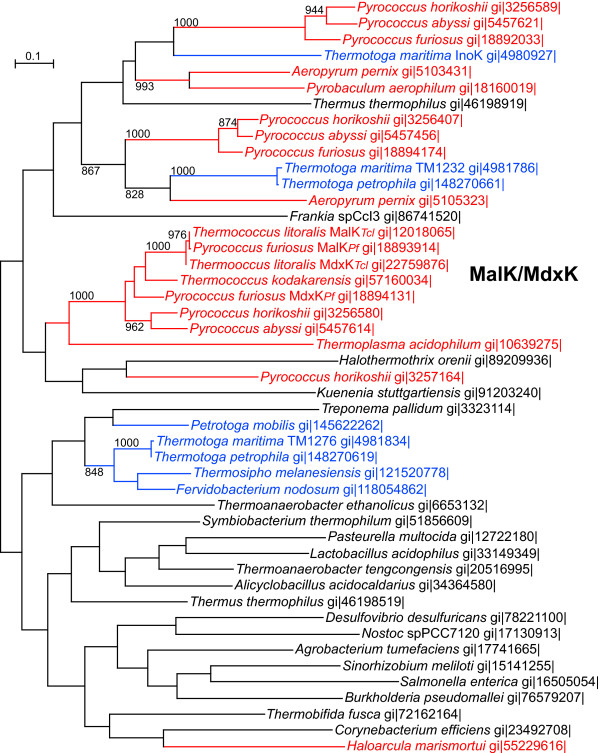
**Maximum likelihood analysis of *P. furiosus *MalK homologs (ATP binding proteins)**. Homologs of the *P. furiosus *MdxK (PF1933) were gathered using that sequence as query in BLASTP. A ML tree using an alignment of these sequences is shown here. Only bootstrap support values over 800 are shown. For reference, the gi numbers of the MalK homologs are provided. Archaeal sequences are shown in red, those from *Thermotogales *in blue, and from other bacteria in black. The tree depicted here should be considered unrooted.

The archaeal *malK *genes have undergone a very different evolutionary history than those in their adjoining *mal *operons. We examined the relationships among *malK *homologs using both MrBayes and PHYML and both gave similar, though not identical trees, each with overall relatively weak support. However, within those trees, clusters of strong support were found and these provide reliable information about the evolutionary relationships within those clusters. As shown in the PHYML-derived phylogeny in Figure 3, all the *Thermococcales malK *and *mdxK *homologs group together in a well-supported clade. This supports the hypothesis that an ancestral *malK2*_An _homolog either was duplicated in an ancestral *Thermococcales *or was horizontally acquired by that ancestor from another *Thermococcales*. The *malK2*_An _was later transferred along with the *mal1*_*An *_operon to a *P. furiosus *ancestor (Fig. 1) [18]. In these genomes, these *malK *genes are near, but not immediately adjacent to, the three-gene *mal/mdx *ABC operons, each encoding two MSPs and one SBP. An amylopullulanase gene typically separates the *malK *homolog from the other genes, but some of these clusters have more genes in the intervening region, perhaps indicative of other HGT or deletion events in some species.

Since the *Tt. maritima mal *operons do not contain adjacent *malK *homologs, we sought in this tree any indication of the identity of a possible *Thermotoga malK *homolog, perhaps derived from the archaeal *malK *or *mdxK *genes. Unfortunately, only two *Tt. maritima *ORFs appear in Fig. 3 among archaeal sequences and all are distantly related to the *Thermococcales malK/mdxK *cluster. The functions of these archaeal transporters are unknown and since none of their ABP or MSP homologs appeared in the BLAST results using the *P. furiosus *MalE, MalF or MalG sequences as queries, they unlikely to be *mal*-related ABPs. A function of TM0421, one of the *Tt. maritima *ORFs found in the vicinity of these archaeal sequences (though with low bootstrap support), has been suggested. Data has indicated that it is a *myo*-inositol transporter's ABP (InoK) [4]. The function of the other ORF, TM1232, is unknown, though its expression was reported to be upregulated when *Tt. maritima *was grown in co-culture with *Methanocaldococcus jannaschii *[23].

A relatively weakly supported cluster of *Thermotogales *ORFs apparent in Fig. 3 is unrelated to any archaeal ORFs. This cluster includes the *Tt. maritima *ORF TM1276 which, based upon gene expression data, has been suggested to encode a maltose transporter ABP [24,25]. Unfortunately this analysis does not provide additional support for that observation. It does not appear that any archaeal *malK/mdxK *homolog was acquired by ancestors of the currently sequenced species of *Thermotogales*.

### Discovery of a third *mal *operon in some members of the *Thermotogales*

Using the *P. furiosus *MalE sequence as a query, homologs were revealed in the genome sequences from *F. nodosum, Ts. melanesiensis, Tt. petrophila*, and *Tt. neapolitana *but not *Tt. maritima *(Fig. 2). Each of these genomes contains complete *malF *and *malG *genes downstream from this *malE *homolog in the order *malEFG*. For *Tt. petrophila *and *Tt. neapolitana *this reveals a third apparent *mal *operon. *Ts. melanesiensis *and *F. nodosum *have only one other *mal *operon (see below), so this is a second for them. This third operon is closely more related to the *mal *operons of *Tc. litoralis *and *P. furiosus *than are the *mal1 *and *mal2 *operons (Fig. 2). Although in that figure they may appear to be specific relatives of one another, that relationship is not strongly supported by either maximum likelihood or Bayesian analyses.

**Figure 4:**
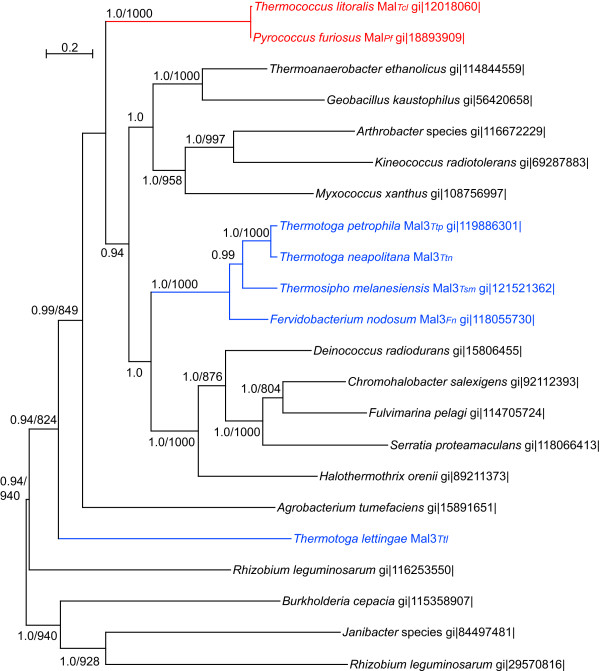
**Relationship among apparent maltose ABC transporters related to the Mal3 homologs found in some members of the *Thermotogales***. Homologs of the *Tc. litoralis *MalE were gathered using that sequence as query in BLASTP. Concatenated sequences of these SBP homologs and their cognate MSP and ABP from each operon were prepared, aligned and analyzed. A ML tree generated in PHYML is shown. Values shown are Bayesian posterior probabilities followed by bootstrap support values calculated with PHYML. Bootstrap support values below 800 and posterior probabilities below 0.8 are not shown. For reference, the gi numbers of the MalE (SBP) sequence for each operon are provided. Archaeal sequences are shown in red, those from *Thermotogales *in blue, and from other bacteria in black. The tree depicted here should be considered unrooted.

To examine the possibility of HGT of these *mal3 *genes between the *Archaea *and the *Thermotogales*, we aligned and analyzed sequences from several additional bacteria in the clade that includes *mal3*. That analysis, shown in Figure 4, does not show a specific association of the *Thermococcales *Mal sequences with these *Thermotogales *sequences, discounting the possibility of HGT of these genes between the *Thermococcales *and *Thermotogales*.

Interestingly, the additional *mal *operon in *Tt. lettingae *(depicted here as "Mal3_*Ttl*_" though it only has two *mal *operons) is not closely related to its homologs in the other members of the *Thermotogales*. It is also not a specific relative of the archaeal *mal *genes. It was likely acquired from a bacterial group different than the donor to the other *Thermotogales*.

### Phylogenetic history of the *Thermotogales mal1 *and *mal2 *genes

Although the *Tt. maritima *Mal paralogs are associated with sequences from enteric bacteria in the *P. furiosus mdxE*-derived phylogeny (Fig. 2), the resolution of that analysis was too low to determine the detailed relationships among the bacterial orthologs. To examine the *Tt. maritima mal1/mal2 *evolutionary history in detail and to determine the relationships among the *mal1 *and *mal2 *genes in the *Thermotogales*, we used the MalE1_*Ttm *_(TM1204) sequence as a query and selected from that dataset sequences from those genes that are arranged in three-gene operons as described previously. Those sequences were concatenated with their MalF and MalG partners. The concatenated sequences from the Mdx_*Pf *_operon was included as an outgroup and all these concatenates were aligned using ClustalX. Trees were constructed using ML and Bayesian analyses as above.

**Figure 5:**
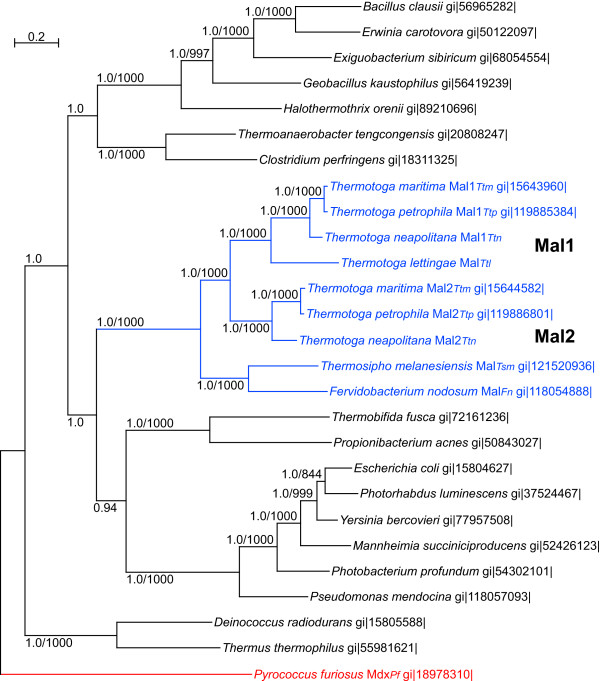
**Relationship among apparent maltose ABC transporters related to the Mal1 and Mal2 homologs found in some members of the *Thermotogales***. Homologs of the *Tt. maritima *MalE1 (TM1204) were gathered using that sequence as query in BLASTP. Concatenated sequences of these SBP homologs and their cognate MSP and ABP from each operon were prepared, aligned and analyzed. A ML tree generated in PHYML is shown. Values shown are Bayesian posterior probabilities followed by bootstrap support values calculated with PHYML. Bootstrap support values below 800 and posterior probabilities below 0.8 are not shown. For reference, the gi numbers of the MalE (SBP) sequence for each operon are provided. The tree is rooted using the Mdx sequences from *P. furiosus *as outgroup, compare with Fig. 2. Archaeal sequences are shown in red, those from *Thermotogales *in blue, and from other bacteria in black.

The resulting phylogenetic tree (Fig. 5) grouped *Thermotogales *sequences together and the relative arrangement of species in this group reflects their branching order as derived using 16S rRNA gene sequence comparisons [3,26]. *Tt. lettingae*, *F. nodosum*, and *Ts. melanesiensis *have only one of this type of *mal *operon while the remaining three *Thermotoga *species each have two of this kind (Fig. 5). These *mal *operons in *F. nodosum *and *Ts. melanesiensis *are related to one another to the exclusion of those from the *Thermotoga *species. The single *Tt. lettingae mal *operon is most closely related to the *mal1 *orthologs. The *mal2 *operon appears to have arisen within the *Thermotoga *lineage either through a duplication of an ancestral *mal1 *or through a horizontal acquisition from an unknown close relative.

An examination of the genomic contexts of these *mal *operons suggests that a simple loss of one of the *mal *operons does not best explain this evolutionary history. As shown in Table 1, the *mal *operons in *F. nodosum *and *Ts. melanesiensis *are not located in an area of the genome that has synteny with any of the other species considered here. This genomic context analysis also supports the observation that this *mal *operon in *Tt. lettingae *belongs to the *mal1 *cluster and that *mal1 *was likely found in the ancestor of all these *Thermotoga *species. We found no region in the *Tt. lettingae *genome sequence that contains a cluster of homologs of the genes surrounding the *mal2 *operon of the other species. Consequently, a simple loss of *mal2 *from the *Tt. lettingae *lineage is not evident. A duplication of *mal1 *after the divergence of *Tt. lettingae *would cause the Mal1 and Mal2 lineages to be exclusively related to one another, but that is not apparent in Fig. 5. Consequently, *mal2 *does not appear to have arisen by a duplication of *mal1*. Rather the data indicate that *mal2 *was acquired by an ancestral *Thermotoga *species from an unknown the *Thermotoga *species after the divergence of *Tt. lettingae*.

**Table 1:** Arrangements of ORFs in the vicinities of the *mal1 *and *mal2 *gene clusters in selected species of the *Thermotogales*. ORFs from different genome sequences are shown here as their best BLASTP hit in the genome sequence of *Tt. maritima*^1^.

	*Tt. maritima*	*Tt. petrophila*	*Tt. neapolitana*	*Tt. lettingae*	*Ts. melanesiensis*	*F. nodosum*
*mal1*	*TM1192*	TM0972				
	*TM1193*	TM1044^2^				
	*TM1194*	*TM1190*				
	*TM1195*	*TM1191*				
	TM1196	*TM1192*				
	TM1197	*TM1193*				
	TM1198	*TM1194*	TM0431			
	TM1199	*TM1195*	TM0810	*TM1193*		
	*TM1200*	*TM1200*	*TM1200*	*TM1200*		
	*TM1201*	*TM1201*	*TM1201*	*TM1201*		
	**TM1202**	**TM1202**	**TM1202**	**TM1202**		
	**TM1203**	**TM1203**	**TM1203**	**TM1203**		
	**TM1204**	**TM1204**	**TM1204**	**TM1204**	TM1578	
	*TM1205*	TM1217	*TM1205*	TM0372	X	
	*TM1206*	TM1218	*TM1206*	X	*TM1581*	
	*TM1207*	TM1219	*TM1207*	TM0945	TM0009	TM1875
	*TM1208*	TM1220	*TM1208*		TM1851	*TM1581*
	*TM1209*	TM1221	*TM1209*		**TM1836**	**TM1836**
					**TM1203**	**TM1203**
					**TM1204**	**TM1839**
*mal2*	TM1829	TM1681			TM1391	
	TM1830	TM1678			X	TM0781
	TM1831^2^	TM1679			X	X
	*TM1832*^2^	TM1677^2^	TM1826		TM1816	X
	*TM1833*	*TM1832*^2^	TM1827		TM1416	X
	*TM1834*	*TM1833*	TM1828		TM1417	TM1103
	*TM1835*	*TM1834*	*TM1834*			TM0690
	**TM1836**	*TM1835*	*TM1835*			
	**TM1837**	**TM1836**	**TM1836**			
	**TM1838**	**TM1203**	**TM1203**			
	**TM1839**	**TM1839**	**TM1839**			
	*TM1840*	*TM1840*	*TM1840*			
	*TM1841*	*TM1841(split?)*	*TM1841*			
	*TM1842*	X	*TM1842*			
	*TM1843*	X	*TM1843*			
	*TM1844*	*TM1844*	*TM1844*			
	*TM1845*	*TM1845*	*TM1845*			

Bold, transporter ORFs; Italics, has at least one homolog in this region in another genome; X, an ORF with no close BLASTP hits in the *Tt. maritima *genome sequence.

^1^Note that all *malF *BLASTP searches only hit TM1203 because TM1838 and TM1837 are pseudogenes in the database.

^2^transposase

With the exception of *Tt. maritima *all species examined here have intact *malF2 *genes. *Tt. maritima *has a *malF2 *pseudogene. Our analyses cannot determine whether the mutations in *malF2*_*Ttm *_occurred during laboratory cultivation of this strain or prior to its isolation from nature.

### MalF transmembrane topologies in the *Thermotogales *and enteric bacteria are similar

Sequence comparisons showed that the *Thermotogales *cluster of Mal1 and Mal2 proteins are specifically related to those from enteric bacteria, so we sought other evidence to support this unusual evolutionary association of proteins from such different kinds of bacteria. We examined putative structural features of the MalF and MalG membrane proteins by hydropathy analyses using the TMpred program [27].

**Figure 6:**
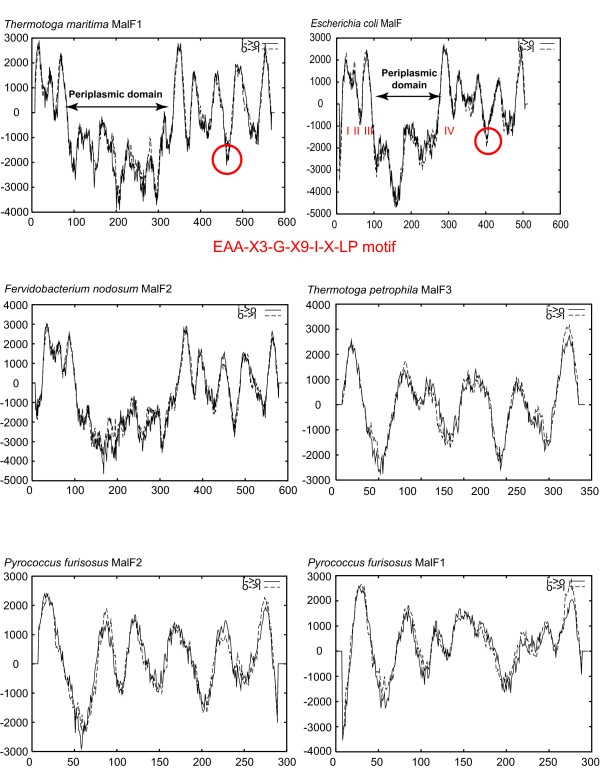
**Transmembrane helices of *Tt. maritima *MalF1 and *Tt. petrophila *MalF3 homologs as predicted by TMpred**. Plots derived from TMpred [27] are shown. In selected proteins the locations of the proposed hydrophilic periplasmic domains and the EEA motifs (circled) are shown. The MalF_*Ec *_is thought to interact with the MalG_*Ec *_motif to promote MalK association with the permease complex [42].

The MalF_*Ec *_is a member of the CUT1 (carbohydrate uptake) family [28] and is one of the most extensively studied membrane permeases [29]. Unlike other membrane permeases, MalF_*Ec *_is unusual in that it consists of eight transmembrane helices instead of six and has a large periplasmic domain of 180 amino acids between transmembrane helices 3 and 4 (Fig. 6) [30]. This peculiar membrane topology is reportedly conserved in all MalF homologs from bacteria closely related to *E. coli *[30]. The function of this domain is unknown, but mutational alterations in this loop affect the localization of MalK_*Ec *_to the membrane bound MalF_*Ec *_[29].

To determine if the MalF topology found in *E. coli *is conserved in the MalF homologs from its close relatives, including members of the *Thermotogales*, transmembrane topological analyses of these sequences were performed. Representative data from those TMpred analyses are shown in Figure 6 and Additional file 1. Both MalF1 and MalF2 homologs from all members of the *Thermotogales *show the eight transmembrane helices, large transmembrane loop, and ABP-interaction motif found in MalF_*Ec*_. These unusual features support the close relationship between the *Thermotogales *MalF1 and MalF2 homologs and those from *E. coli *relatives. The phylogenetic analyses reported above are not influenced by the presence of this large loop since tree topologies are not changed when these loops were removed from the sequences prior to alignment (data not shown).

The MalF homolog from *Propionibacterium acnes*, also related to the *E. coli *MalF, has a smaller, but still evident, loop (see Additional file 1). The conservation of this unique large loop in the MalF permeases in these distantly related organisms is consistent with their shared evolutionary history. Interestingly the MalF from *Deinococcus radiodurans *(and *Thermus thermophilus*, not shown) has a smaller loop while *Thermoanaerobacter tengcongensis *MalF (and those closely related to it) show no evidence of a loop (see Additional file 1). Whatever its function, this loop is more pronounced in close relatives of the enteric form of MalF and was apparently lost from the largely gram positive clade of organisms.

The MalF3 homologs found in some members of the *Thermotogales *and in *P. furiosus *and *Tc. litoralis *do not contain this loop feature (Fig. 6). This observation supports the large evolutionary distance between the *mal3 *genes and the *mal1-mal2 *genes.

### Unusual domain architectures of MalG in the *Thermotogales*

**Figure 7:**
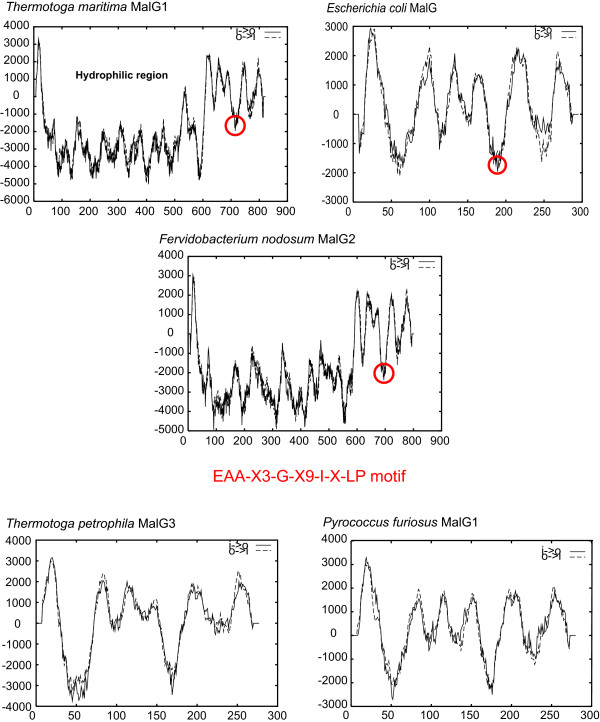
**Transmembrane helices of *Tt. maritima *MalG1 homologs as predicted by TMpred**. Plots derived from TMpred [27] are shown. Solid and dashed lines depict inside to outside and outside to inside orientations of the helices predicted by TMpred, respectively. The location of the proposed hydrophilic region in the *Tt. maritima *MalG1 and the EEA motifs (circled) are shown. This motif is involved in MalK binding in the *E. coli *maltose transporter [42].

The MalG1 and MalG2 homologs in the *Thermotogales *are unusually large as compared to their homologs from other bacteria including *E. coli *(Fig. 7). The alignments of MalG homologs showed that the C-terminal 300 amino acids of MalG1_*Ttm *_and MalG2_*Ttm *_are similar to the MalG homologs from *E. coli *and related bacteria, but the N-terminal 525 amino acids showed no significant sequence similarity to any known proteins. TMpred analyses revealed a large hydrophilic region of about 500 amino acids between transmembrane helices one and two of these MalG1 and MalG2 sequences (Fig. 7). The MalG3 homologs lack this region, supporting their phylogenetic placement relative to Mal1/Mal2 (Fig. 7).

## Discussion

Members of the archaeal Order *Thermococcales *have participated in intradomain lateral transmission of ABC transporter genes. This is most clearly seen in the sharing of maltose/trehalose transporter genes [8,18]. The genome of the bacterium *Tt. maritima *has a disproportionate representation of ABC transporters, many of which appear to have been acquired from archaea [3,31]. Since *Tt. maritima *has two *mal *operons and lives among species of the *Thermococcales*, we examined the evolutionary history of its *mal *genes to look for evidence of possible interdomain HGT.

Our analyses show that the *mal *operons found in the currently sequenced *Thermococcales *genomes have undergone a complex evolutionary history. We confirmed the earlier suggestions that *P. furiosus *acquired its *mal *operon from an ancestor of *Tc. litoralis *and that that operon acquired its *malK *homolog in an ancestor of *Tc. litoralis *from an ancestral *mal2 *operon. There is insufficient information to determine whether *malK2*_An _was acquired by the ancestral *mal1 *operon from another organism *via *HGT or by gene duplication from the same chromosome.

We found that the ancestor of the *Thermococcales mal *genes was clearly in the bacterial lineage. Analysis of concatenations of MalE, MalF, and MalG sequences show the *Thermococcales *Mal proteins are most closely related to bacterial homologs. In contrast, the Mdx sequences cluster separately in a distant archaeal lineage. Since *P. abyssi *and *Tc. kodakarensis *do not contain a second *mal *operon, the *mal1*_An _genes must have been acquired in the *Tc. litoralis *lineage.

The ancestors of the *Thermococcales mal *genes were acquired from a bacterial lineage relatively distant from that that gave rise to the two *mal *operons in *Tt. maritima*. However, several other members of the *Thermotogales *have a *mal *operon (in some of these it is a third *mal *operon) that is from the same bacterial lineage as the *Thermococcales mal *operons. We found no evidence that this operon was directly transferred between the *Thermococcales *and *Thermotogales *lineages, though. Sequence comparisons cannot demonstrate the functions of these newly revealed *mal *operons. Investigations into the binding properties of their SBPs are underway to elucidate their potential physiological roles.

The genome sequence of *Tt. maritima *strain MSB8 published by The Institute for Genome Research in 1999 did not contain a third *mal *operon. Prior to that publication, in 1993, W. Liebl deposited in GenBank the sequence of a *Tt. maritima *strain MSB8 gene encoding a β-glucosidase (*bglA*) that was contained on a cloned fragment that also contained a portion of an apparent *malE *gene upstream of this *bglA *[32]. Neither this *bglA *gene nor the adjacent *malE *appeared in the subsequent TIGR genome sequence. This suggests that after the strain was deposited in the DSMZ strain collection, it may have suffered a deletion of its *mal3-bglA *region. We are investigating this possibility using cultures of the type strain and the strain used for genome sequencing.

The modern *Thermococcales *MalK homologs are descended from an ancestral MalK2. Since all the examined archaeal *mal/mdx *operons have a nearby *malK/mdxK *gene, all of which are closely related to one another, we cannot know if the ancestral *mal1 *operon inherited from the bacteria contained a *malK *gene or not. Despite the relatively close relationship of the *Thermococcales *Mal sequences to those of the *Thermotogales *Mal3 sequences, there are no obvious orthologs of the archaeal MalK/MdxK in any of the *Thermotogales*. The membrane components of ABC transporters are known to recruit different ABPs to effect transport, so the lack of a *malK *near these *Thermotogales *operons is not unusual. Two *Tt. maritima *ORFs, TM1276 and TM1232, were identified here as potential MalK homologs by phylogenetic analyses, but we did not observe up regulation of either ORF in response to growth on maltose [33] though there is a report that TM1276 is expressed in response to growth on maltose [25].

Sequence comparisons of the *Thermotogales *Mal1 and Mal2 concatenates demonstrated their close relationship to one another. Three of the examined members of the *Thermotogales *have only one member of this family of *mal *operons. Gene synteny comparisons and phylogenetic analyses suggest that *mal1 *was present in the ancestor of *Tt. maritima*, *Tt. petrophila*, and *Tt. neapolitana *and that *mal2 *entered that ancestor by HGT from an unknown *Thermotoga *species. *F. nodosum *and *Ts. melanesiensis *both have a single *mal *operon of the *mal1/mal2 *type, but the genetic contexts of those operons are unlike those found in any of the other organisms. Their sequences do not place them uniquely with either the *mal1*- or *mal2*-type genes, a situation one might expect for ancestral-type sequences. It will be very interesting to determine the functions of these transporters from these two species to compare them with the evolved Mal1 and Mal2 transporters.

The *Thermotogales *Mal1 and Mal2 sequences consistently clustered in a lineage that included the Mal sequences from the gamma proteobacteria. The relationships revealed in the Mal1/Mal2 phylogeny are supported by the phylogenetic distribution of a unique secondary structure of the MalF homologs. All the MalF homologs with eight transmembrane helices and a large periplasmic loop between transmembrane helices three and four are clustered in our phylogenetic analysis (Fig. 5). A large unique hydrophilic region in the *Thermotogales *MalG1 and MalG2 proteins confirms their close evolutionary relationship with one another and indicates a relatively recent acquisition of this domain. This region is not found in any other MalG protein and its function is as yet unknown.

## Conclusion

We confirmed earlier suggestions that *P. furiosus *acquired its *mal *operon from an ancestor of *Tc. litoralis *and that that operon acquired its *malK *homolog in an ancestor of *Tc. litoralis *from an ancestral *mal2 *operon. Our analyses show that the ancestor of the *Thermococcales mal *genes was in the bacterial lineage while the ancestor of the *mdx *genes was from an archaeal lineage. The bacterial lineage from which came the *Tc. litoralis mal *operon also gave rise to a newly discovered *Thermotogales mal *operon, the third in some extant *Thermotoga *species. We find no evidence that the archaeal *mal *genes or the *Thermotogales mal3 *genes were shared between these groups.

The *Thermotogales *Mal1/Mal2 sequences consistently clustered in a lineage that included the Mal sequences from the gamma proteobacteria. The appearance of paralogous *mal *operons in some *Thermotoga *species took place by acquisition of an orthologous *mal2 *operon by HGT.

The relationships among bacterial *mal *genes revealed in the Mal1/Mal2 phylogeny are supported by the phylogenetic distributions of unique secondary structures in the MalF proteins. The enteric bacteria and the *Thermotogales *contain MalF homologs with eight transmembrane helices and a large periplasmic loop between transmembrane helices three and four cluster. A unique large hydrophilic region in the MalG proteins from the members of the *Thermotogales *confirms their close evolutionary relationship to one another and indicates a relatively recent acquisition of this region.

## Methods

### Data acquisition

Protein sequences were retrieved by BLASTP searches of the nonredundant protein database at NCBI [34] using as queries amino acid sequences of *Tt. maritima*, *P. furiosus*, or *Tc. litoralis *MalE, MalF, or MalG. The *P. furiosus *MalK2 (PF1933) was used as the query to search for MalK homologs among bacteria and archaea. The top 250 sequences retrieved by BLASTP searches were assembled into datasets and repeated sequence entries and closely related sequences (determined using neighbor-joining trees of aligned sequences) were removed. Those sequences from three-gene operons (*malE*, *malF *and *malG*) were retained and the cognate genes were concatenated. Alignments of the amino acid sequences of these concatenates were prepared using ClustalX v1.83.1 [35].

TM1837, the *malF2*_*Ttm *_ORF, contains an authentic frameshift mutation. To make comparisons between MalF homologs, the pseudogene was corrected by removing nucleotides at positions 254 (A) and 298 (G) (identified using GENIO/frame [36]). Corrections at these positions restored the reading frame without introducing new stop codons. *malF2*_*Tm *_is preceded by a small ORF, TM1838. The putative 54 amino acid peptide product of *TM1838 *is 53% similar to the amino terminus of MalF1_*Ttm*_. By adding a nucleotide between positions 138 and 139 in the TM1838 sequence and combining it with the corrected *malF2*_*Ttm*_, a single polypeptide is obtained that has a high similarity to *malF1*_*Tm *_as measured by PRSS (P value 1.6 e-21) [37]. It appears that a series of point mutations in an ancestral *malF2 *created TM1838 and the translationally truncated *malF2*_*Ttm*_. Consequently, there is no functional MalF2_*Ttm *_protein produced by *Tt. maritima*.

### Phylogenetic analyses

Aligned sequences from the concatenate datasets were used to construct consensus trees using MrBayes v3.1 [38] and PHYML v2.4.4. The MrBayes analyses were performed using the default GTR model and gamma distributed rate variation with two runs, each with four chains, for 1,000,000 generations (780,000 for the tree shown in Fig. 2) and taking a consensus tree after a burn-in of either 100,000 or 65,000 generations. These analyses were performed at the Bioinformatics Facility of the University of Connecticut Biotechnology Bioservices Center. Maximum likelihood analyses of these alignments were performed in PHYML with 1000 bootstrap resamplings, the JTT substitution model, a fixed proportion of invariable sites, one category of substitution rate, and the BIONJ input tree.

### Other sequence analyses

The analyses above were confirmed with alternative alignments using MUSCLE [39] and T-COFFEE [40] each using their default parameters. These analyses produced phylogenies nearly identical to those shown here when used with PHYML (WAG model, estimated pinvar, estimated gamma distribution) (not shown). Transmembrane topological analyses were performed using TMpred [41,27].

## Authors' contributions

DMN conceived the project; conducted preliminary phylogenetic and protein domain analyses; and contributed to the writing of the manuscript.

PL assisted in the compilation and analyses of the sequences and reviewed the manuscript.

JPG assisted in the data analyses and reviewed the manuscript.

KMN oversaw the project; compiled datasets and executed their analyses; analyzed protein domains; and was primary author of the manuscript.

## Supplementary Material

Additional file 1 Transmembrane helices of *Tt. maritima *MalF1 bacterial homologs as predicted by TMpred. Plots derived from TMpred [27] are shown. Solid and dashed lines depict inside to outside and outside to inside orientations of the helices predicted by TMpred, respectively. Click here for file
